# Occurrence of *Blastocystis* in Water of Two Rivers from Recreational Areas in Malaysia

**DOI:** 10.1155/2011/123916

**Published:** 2011-06-06

**Authors:** Init Ithoi, Azman Jali, J. W. Mak, Wan Yusoff Wan Sulaiman, Rohela Mahmud

**Affiliations:** ^1^Department of Parasitology, Faculty of Medicine, University of Malaya, 50603 Kuala Lumpur, Malaysia; ^2^Department of Biology, Faculty of Science, Putra Malaysia University, 43400 Serdang, Selangor, Malaysia; ^3^School of Postgraduate Studies and Research, International Medical University, Bukit Jalil, 57000 Kuala Lumpur, Malaysia

## Abstract

This study reports the occurrence of *Blastocystis* in water from two rivers, Sungai Congkak and Sungai Batu, located in recreational areas in Malaysia. This protozoan was detected in samples from both rivers with an average of 33.3% and 22.1%, respectively. It was detected highest at the downstream (73.8% and 33.8%) followed by midstream (17.5% and 25.0%) and upstream (8.8% and 7.5%) stations, with additionally higher detection during holidays (with average 47.5% and 30.8%) than week days (with average 19.2% and 13.3%), in both rivers, respectively. There was a strong association with the daily activities of locals and visitors, who came for water recreational activities, mainly located between midstream and downstream and was observed to be higher at Sungai Congkak. The detection of *Blastocystis* in these rivers' water implies that this protozoan could potentially be transmitted to humans by the waterborne route. Pearson correlation analysis showed that their occurrence was significantly correlated with faecal coliforms count; inconsistent correlation with dissolved oxygen, temperature and turbidity and no correlation with pH, conductivity and rainfall for both rivers. The correlation of coliforms and *Blastocystis* suggests the source of the *Blastocystis* in the water body is likely to be faecal.

## 1. Introduction

*Blastocystis *has been associated with human gastrointestinal disease in both immunocompromised and immunocompetent hosts [1, 2]. Clinical features due to infection by this protozoan include acute or chronic diarrhea, sometimes self-limited, and could be accompanied by abdominal pain, tenesmus, pruritus, anorexia, nausea, vomiting, fever, and eosinophilia [3, 4]. The symptom is generally more severe in immunosuppressed patients than in immunocompetent hosts [5, 6]. *Blastocystis* is globally distributed and infections are common in tropical, subtropical, and developing countries [7]. Other than food, contaminated water was reported to be an important risk factor for *Blastocystis* transmission via the faecal-oral route [8, 9]. In Malaysia, although *Blastocystis* infection has attracted the attention of many researchers, studies on its contamination of surface waters are still lacking. To date, there is no published information on its occurrence in recreational water sites even though this has important implications to the public health. Therefore, this study was carried out to determine the occurrence of *Blastocystis* in two rivers which are widely used for recreational activities amongst people from the capital city of Kuala Lumpur, Malaysia. Subsequently, its occurrence was correlated with physical and biological factors of the river water as well as rainfall and human activities in the surrounding areas.

## 2. Materials and Methods

### 2.1. Sampling Sites and Collection of Water Samples

**Figure 1:**
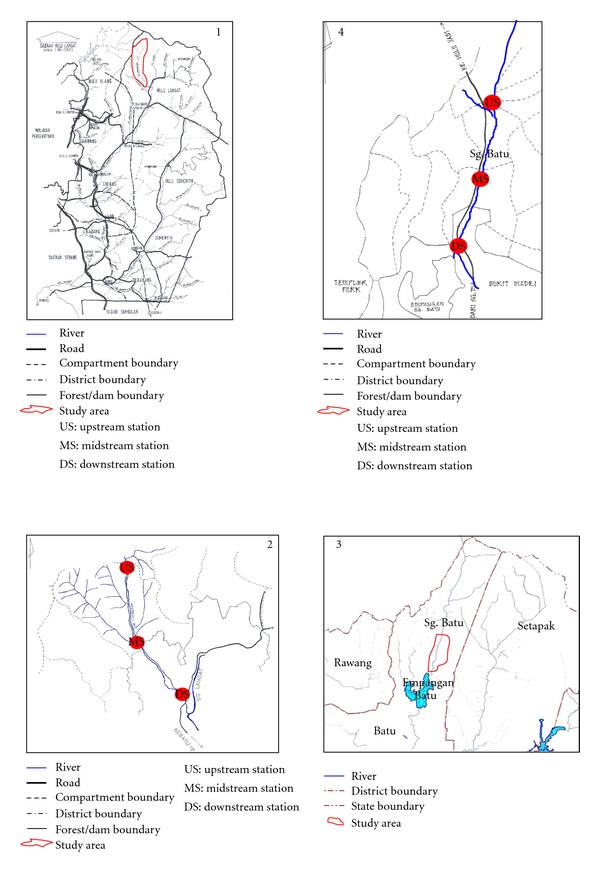
Map showing the locations of sampling stations at study area of Sungai Congkak (1 and 2) and Sungai Batu (3 and 4), Selangor, Malaysia.

The water samples were collected from two recreational rivers, Sungai Congkak and Sungai Batu, located in Selangor state, Malaysia (Figure 1). At each river, 3 sampling stations—upstream, midstream, and downstream—with a distance of 3000 meters between each station were selected. The upstream station is located at secluded areas and the water is naturally clean. The midstream station is located at the beginning of the Malaysian Aborigines (Orang Asli) settlement. The downstream station is located about 500 meters after human settlement area. The recreational activities were carried out at about 1000 meters from upstream, and further down to about 500 meters before downstream station. The surface water from each station was collected once a month for 10 months continuously, starting from August 2004 to May 2005. Of these 10 visits, 5 visits were carried out during the week days and 5 visits during weekends (or public holidays). Water samples were collected from each station in sterile 500 mL and 1000 mL Schott bottles. The water samples in 500 mL bottle (from the middle of the rivers) were placed in a cold ice-packed box (for isolation of faecal coliforms) while those in 1000 mL bottle (one each from the middle and side of the rivers) were placed in a normal box (for isolation of *Blastocystis*), and transported as soon as possible to the laboratory. They were then processed on arrival or within 8 hours after sampling.

### 2.2. Measurement of the Water Physical Parameters

Five physical parameters of water which were turbidity, temperature, conductivity, pH, and dissolved oxygen were measured *in situ* during every sampling activity, using a turbidity meter (ICM, Oregon, USA, model 2100P), multipurpose meter (Yellow Springs Instrument Co. Ins., Ohio, USA, YSI model 33; for temperature, pH, and conductivity), and oxygen meter (model YSI 58), respectively. The parameter values obtained from each sampling activity were recorded and accumulated for the correlation analysis with the number of *Blastocystis* positive culture plates.

### 2.3. Measurement of the Rainfall Data

Rainfall data from two rainfall stations were obtained from the Department of Meteorology, Petaling Jaya, Selangor, Malaysia. These stations were the Ulu Langat Dam and Ulu Gombak, which are the nearest to the Sungai Congkak and Sungai Batu, respectively.

### 2.4. Determination of the Local Community and Recreational Visitors

The number of local people who lived around these two rivers, and visitors who came during the week days and public holidays for water-related recreational activities were obtained by personal observation during sampling visits, and by personal communication with the “penghulu” (head of the local community) and forest officers (officials from Selangor Forestry Department who monitor forest reserve areas) of respective recreational water areas.

### 2.5. Cultivation and Detection of Blastocystis

**Figure 2:**
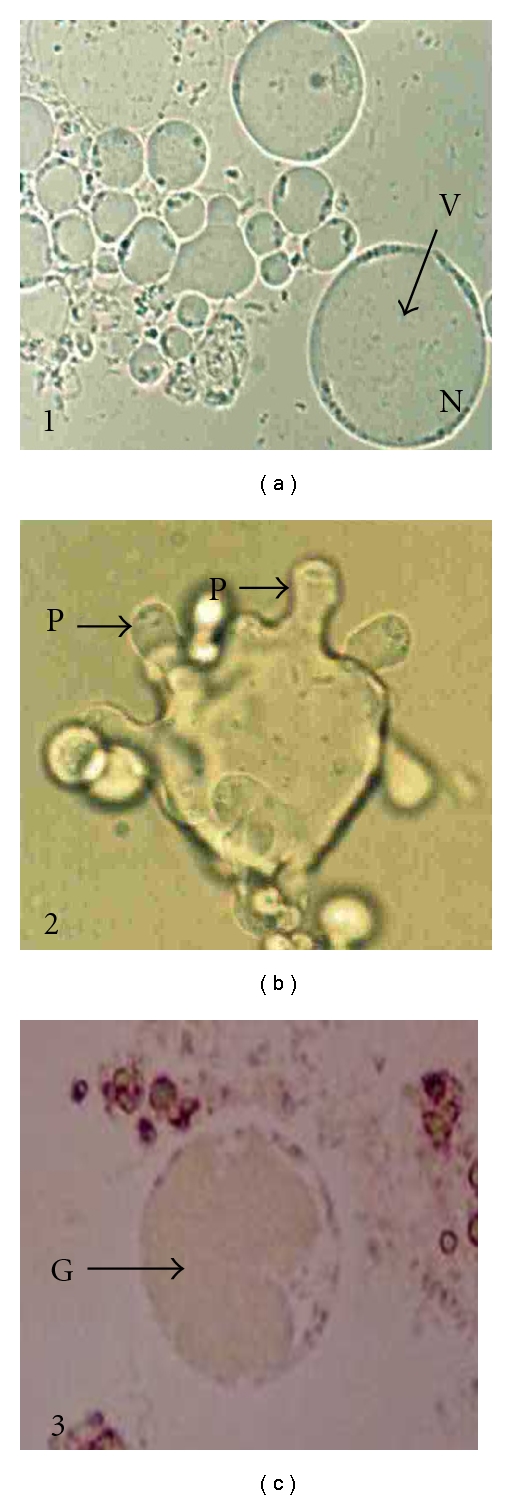
Blastocystis cells (×400) from water samples showing the vacuolar (1), amoeboid (2), and granular (3) forms. V: vacuole, N: sandy nuclei, P: pseudopodia, G: vacuole with granules.

A thousand (1000) mL of each water sample was filtered through a cellulose ester membrane (47 mm diameter, 1.2 *μ*m pore size, Millipore, Bedford, USA) held in a filter holder. The sediment on the membrane was collected by thoroughly rinsing with sterile distilled water using a plastic pipette. The residual water was then transferred into a 50 mL centrifuge tube and centrifuged at 684.2 ×*g* (Kubota Corporation, Japan, Kubota model 2010) for 15 minutes. The supernatant was discarded by using sterile pipette until 5 mL was left. The pellet was then resuspended and 5 drops were inoculated into a 1.5-mL eppendorf tube containing 1 mL complete Jone's medium [1]. Four replicates of inoculation were carried out for each water sample. Each of positive and negative control was also carried out in 4 replicate tubes by inoculation with *Blastocystis* and distilled water respectively. These inoculated tubes were tightly closed, placed in a rack, and incubated at 37°C. The medium in each of these tubes was replaced with the new complete Jone's medium every alternate day starting from day 2 of cultivation. This was carried out by discarding about 800 *μ*L of the medium at the top level (without disturbing the pellet) and replaced by adding 800 *μ*L of new complete Jone's medium. The presence of *Blastocystis* was observed daily for 14 days of cultivation, by placing 1 drop of cultured sediment onto a glass slide, covered with a cover-slip, and viewed (×100 and ×400 objectives) under light microscopy (Olympus BX51). *Blastocystis* cells were seen as vacuolar, sizes 4–15 *μ*m (common), amoeboid (rare in some culture tubes), and granular (many in old culture tubes), forms as shown in Figure 2. The number of positive tubes for *Blastocystis* was then recorded.

### 2.6. Cultivation and Calculation of Faecal Coliforms

Faecal coliforms count was performed according to a method published by Anonymous [10]. Briefly, the bottle was shaken to resuspend the water sample and then sieved with sterile stainless sieve to separate from large particles. Only 100 mL of this sieved water sample was used in this experiment while the rest was kept (as stock) in 4°C for repeat experiment (if necessary). Each of 50 mL (out of 100 mL) was further filtered through a cellulose ester membrane (0.45 *μ*m pore size, 47 mm diameter, Millipore), fitted on its glass filter holder (Nalgene), using a vacuum pump. Filtered membrane (with debris) was carefully lifted and placed on top of the surface of a lauryl sulphate membrane (Oxoid) which was first placed on the sterile plate. The plate was closed, sealed with parafilm, and incubated at 30°C for 4 hours to resuscitate injured bacteria before they were further incubated in a 44.5°C incubator for 14 hours. Faecal coliforms (yellow colonies) were counted and recorded as mean (of two replicate plates) colony forming unit per 100 mL (CFU/0.1 L). For those samples with heavy colonies (or uncountable), this experiment was repeated by using 100 mL of similar water sample from the stock, diluted (between 0.1 to 1.0 dilution) with sterile Ringer's solution (Oxoid) before filtration.

### 2.7. Statistical Analysis

The linear regression analysis was carried out by using a statistical software package (SPSS version 11.5).

## 3. Results

### 3.1. Physical Parameters Data

The ranges of dissolved oxygen (DO), pH, temperature, conductivity, and turbidity of water from the upstream, midstream, and downstream stations of Sungai Congkak were 7.92–9.30 mg/L, 6.20–7.10, 22.10–27.40°C, 26.60–56.40 *μ*S/cm, and 1.90–14.00 NTU; Sungai Batu were 7.80–9.34 mg/L, 6.29–6.99, 22.00–24.70°C, 23.00–42.00 *μ*S/cm, and 1.60–183.00 NTU (Table 1, A and B).

### 3.2. Rainfall Data

The monthly rainfall measurements at these stations are shown in Table 2. The average volume during the 10-month period (August, 2004 to May, 2005) for both rainfall stations was almost similar, with the mean ± SE of 181.22 ± 86.28 mm and 184.06 ± 73.63 mm at Ulu Langat Dam (near to Sungai Congkak) and Ulu Gombak (near to Sungai Batu), respectively. The driest month was on January, 2005, and the most rainfall was in November 2004.

### 3.3. Number of the Local People and Recreational Visitors

Local settlement was more crowded along the Sungai Congkak, with about 1000 houses and more than 3000 residents while at Sungai Batu, there were about 300 houses with less than 1000 people. The average number of visitors on week days was only around 10–20 people at both rivers while on holidays the average number increased dramatically to around 1500–2000 people at Sungai Congkak and 1000–1500 people at Sungai Batu. These visitors mostly did their recreational activities along the rivers between upstream and downstream stations with the greatest number packed around midstream.

### 3.4. Number of Culture Tubes with Blastocystis Spp. and Faecal Coliforms Count

Culture tubes with positive *Blastocystis *and faecal coliforms count were mostly detected in the water samples collected from downstream, followed by midstream and upstream stations. Faecal coliforms were detected in all the water samples. *Blastocystis,* was detected in selected water samples collected from the side and middle of the river. Both *Blastocystis* and faecal coliforms were detected more frequently and higher during holidays than weekdays, and in Sungai Congkak than Sungai Batu (Table 3).

### 3.5. Correlation of Blastocystis with Faecal Coliforms, Physical Parameters, and Rainfall

For Sungai Congkak, the occurrence of *Blastocystis* showed significantly correlation with faecal coliform counts (*r* = 0.80, *P* < .01) and temperature (*r* = 0.76, *P* < .01). In Sungai Batu, it showed correlation with faecal coliforms count (*r* = 0.50, *P* < .01), dissolved oxygen (*r* = 0.37, *P* < .05), and turbidity (*r* = 0.40, *P* < .05). There was no significant correlation with pH, conductivity, and rainfall in both rivers (Table 4).

## 4. Discussion

Drinking contaminated water is known to be one of the important factors for *Blastocystis* infection [11, 12]. From our knowledge, there was no information on the presence of this protozoan in surface waters in Malaysia as well as other regions. *Blastocystis* was not listed as a waterborne protozoan pathogen [13] although there were early reports that this protozoan was transmitted via the fecal-oral route through contaminated water and food [8, 9]. In the current study, we selected two rivers, Sungai Congkak and Sungai Batu, as their raw water has been frequently consumed by most of the local communities and visitors that came for recreational activities. Most of these people are either, innocent, unaware, or lacked knowledge (especially children) about waterborne protozoan pathogens in the water of these rivers. Two important pathogenic water-borne protozoa, *Giardia* and *Cryptosporidium* species, were previously reported to contaminate water of these rivers [14]. While in this study, another water-borne protozoan, *Blastocystis* species was detected with higher percentage in the water samples collected from Sungai Congkak (33.3%; 80/240) than Sungai batu (22.1%; 53/240). It was detected in water from all three sampled stations (up-, mid-, and downstream), in which its occurrences in each station was correlated with faecal coliforms, thus associated with the activities of local community (human and animals) and visitors who contributed to faecal contamination. Subsequently, *Blastocystis* cysts were also widely distributed at the water surface and river beds since it was also detected in water from the side and middle parts of the rivers. 

The upstream stations of both rivers are located at secluded areas with no human habitation and the water is naturally clean. It is near to the natural source of water, originating from the hill (water catchment area) and is less exposed to any form of faecal contaminant except from wildlife in the surrounding jungle. At the upstream of Sungai Congkak, *Blastocystis* was detected during holidays (weekends and public holidays) and none was found during weekdays, therefore, we suspected that the source for this protozoan was probably from infected humans (local or visitors) who defaecated within this area of the river. However, in Sungai Batu, this protozoan was detected during holidays and weekdays, thus suggesting that the sources of contamination were from humans as well as wild animals in the surrounding area. During water sampling visits, we observed wild animals such as wild boars, oxen, tapir, and porcupine at this area. The faeces of these wild animals could be a source for *Blastocystis* since this protozoan is a common parasite in most animals [15].

The midstream station, which was located about 3000 meters from the upstream, was the main source of contamination at both rivers. Human settlements were located here. There were houses without water facilities and proper toilets. There were visitors who picnicked at these areas during weekends or on public holidays. Several activities such as agriculture (vegetables planting and livestock breeding for family consumption) were carried out between the midstream and downstream, and recreational areas between lower part of upstream and upper part of downstream. These were believed to be strongly associated with the increment of *Blastocystis* occurrence at these stations of both rivers. On the other hand, the tributaries which joint the rivers at the upper part of the midstream, could also contribute *Blastocystis* in the water at the midstream stations. These tributaries (very small brooks) were free from human habitation and activities, in which their environments were similar as at the upstream areas, thus feral animals were the most possible source that may contribute *Blastocystis* contamination in these tributaries. 

Downstream station of both rivers showed the highest contamination with *Blastocystis* and faecal coliforms, thus may be due to the dislodgement of *Blastocystis* cysts that were sedimented at the river bed, through activities by the users (bathers and swimmers, especially during holidays), causing them to float downstream. In addition, we had seen children's disposable diapers with faeces and rubbish accumulated at these areas. Animals such as cats, dogs, and crows were also wandering at these areas of the rivers.

Sungai Congkak has larger human settlements, agriculture activities, and visitors who came for recreational water-related activities during holidays. The houses of local people were also seen more crowded along this river as compared to those in Sungai Batu which were scattered further apart from each other. These subsequently may lead to higher incidence of the direct or indirect disposal of infected faeces, sewage, and wastewater, which were the important sources for *Blastocystis* contamination.

Meanwhile during holidays, the occurrence of *Blastocystis* obviously increased (19.6%; 94/480) as compared to week days (8.1%; 39/480), in which it was associated with human water-related activities by visitors, with an average number of around 1500–2000 and 1000–1500 people at Sungai Congkak and Sungai Batu, respectively. In contrast, there were fewer visitors (10–20 people) during the weekdays at both rivers. The water-related activities seen at both rivers were swimming, snorkeling, water sports, camping and barbecue at the river site, jungle trekking from midstream up to lower part of upstream station, and motivation programs (educational visits, teamwork activities), and were actively carried out along the rivers between upstream and downstream, with the greatest number of these visitors (95%) packed around midstream stations. 

As *Blastocystis *is a common intestinal protozoan causing mostly asymptomatic infection in healthy people and these infected people (local and visitors) may have contaminated the river water during their water related activities. Bathers were believed to be important sources of contamination with waterborne pathogens for both treated and untreated recreational waters [16]. Several workers have noted that there were positive relationships between number of bathers and the levels of waterborne *Cryptosporidium parvum *oocysts and* Giardia intestinalis* cysts in recreational water [17, 18], and such relationship also exist for other waterborne protozoan pathogens as well [19]. Additionally, when the *Blastocystis*-infected people, continues to swim in recreational water, this increases the likelihood of contamination of water with this parasite and can result in bather cross-infection [20, 21].

Subsequently, the results from Pearson correlation analysis revealed that only fecal coliform showed significant correlation with the occurrence of *Blastocystis* in the water from both rivers. Other parameters showed inconsistent correlation (e.g., dissolved oxygen, temperature, and turbidity) or no correlation (e.g., pH, conductivity, and rainfall) with the occurrence of this protozoan. Thus faecal coliforms could be ideal assessors of potential *Blastocystis* contaminations in the water body, as coliforms are easier to detect than *Blastocystis*. Therefore, more studies need to be carried out in order to confirm their consistent relationships, since this significant correlation was first to be reported. On the other hand, screening for pathogenic parasites is an important adjunct for the assessment of water quality, because the lack of coliforms does not necessarily imply the absence of pathogenic protozoa [22]. *Blastocystis* may be expected to be present in the river water wherever faecal contamination is detected, since this parasite is a common intestinal protozoan in both man and animals [3]. Furthermore, all the residents in Sungai Batu and majority in Sungai Congkak were not provided with safe water supply facilities by the relevant agencies. Even in the case of local residents who received safe water supply, according to some residents, the water is of low quality and rust colour. For this reason the local people used the river water for their daily activities such as swimming, bathing, washing, and even for drinking and cooking. We had also seen on several occasions local residents defaecating and cleaning up after defaecation in these rivers. Therefore, the possibility is always there for these local people to be infected with *Blastocystis* through consumption of this contaminated water. 

Sungai Congkak and Sungai Batu flowed into the Sungai Langat and Batu Dam, respectively. The presence of *Blastocystis* in these two rivers may present a risk to the public/consumers, because both, Sungai Langat and Batu Dam, are used as sources of water supply for several nearby districts. Cysts of *Blastocystis* are very small and resilient to the standard water chlorination [23]. The cysts are viable for more than 1 or 2 months in distilled water at 25 or 4°C, respectively. Oral inoculation of 10 cysts into each of 10 rats showed 20–100% infectivity, indicating that contamination with only a few cysts can cause *Blastocystis *infection [24]. All these characteristics allow them to persist in the environment for a long period of time, thus potentially exposing public/consumers to infection.

## Figures and Tables

**Table 1:** Mean physical parameters of the water at up-, mid-, and downstream station of Sungai Congkak and Sungai Batu.

Mean ± SD of parameter	Sungai Congkak			Sungai Batu		
	Up-S	Mid-S	Down-S	Up-S	Mid-S	Down-S
DO (mg/L)	8.53 ± 0.15	8.65 ± 0.07	8.39 ± 0.12	8.82 ± 0.18	8.68 ± 0.21	8.72 ± 0.14
pH	6.68 ± 0.13	6.60 ± 0.14	6.61 ± 0.13	6.60 ± 0.10	6.54 ± 0.08	6.54 ± 0.05
Temp (°C)	23.05 ± 0.29	23.43 ± 0.27	25.42 ± 0.51	23.09 ± 0.27	23.52 ± 0.27	23.95 ± 0.33
Con (*μ*S/cm)	35.73 ± 4.50	34.17 ± 3.92	37.10 ± 4.74	28.60 ± 2.67	28.97 ± 2.89	30.9 ± 3.21
Tur (NTU)	3.12 ± 0.46	6.09 ± 2.22	4.96 ± 0.93	22.51 ± 8.21	19.25 ± 6.25	17.66 ± 4.10

Up-S = upstream, Mid-S = midstream, Down-S = downstream, DO = dissolved oxygen, Temp = temperature, Con = conductivity, *μ*S = micron siemens, Tur = turbidity, NTU = nephelometric turbidity unit, SE = standard error.

**Table 2:** Rainfall data at Ulu Langat Dam (near to Sungai Congkak) and Ulu Gombak (near to Sungai Batu) rainfall stations.

Measurement date (month, year)	Rainfall volume (mm) at Ulu Langat Dam	Rainfall volume (mm) at Ulu Combak
August, 2004	69.10	92.70
September, 2004	212.00	253.60
October, 2004	308.20	245.60
November, 2004	522.70	521.40
December, 2004	42.80	36.00
January, 2005	1.60	13.50
February, 2005	34.30	125.00
March, 2005	82.80	163.80
April, 2005	160.80	126.40
May, 2005	377.90	262.60

Mean ± SE	181.22 ± 86.28	184.06 ± 73.63

SE = standard error.

**Table 3:** Number of culture tube containing *Blastocystis* and faecal coliforms count at the up-, mid-, and downstream sampling sites of the rivers.

Sungai Congkak				Sungai Batu			
	No. CuT + B		CFU/0.1L × 10^3^		No. CuT + B		CFU/0.1 L × 10^3^
sampling date (SDate)	SR	MR	MR	SDate	SR	MR	MR

Upstream station							
04-08-04	—	—	0.68	16-08-04	—	—	1.00
**05-09-04**	**—**	**2**	**2.60**	**19-09-04**	**—**	**—**	**0.65**
07-10-04	—	—	0.51	22-10-04	—	—	0.41
**07-11-04**	**—**	**1**	**0.60**	**28-11-04**	**—**	**1**	**0.54**
16-12-04	—	—	0.48	22-12-04	—	—	0.60
**09-01-05**	**3**	**—**	**1.60**	**23-01-05**	**—**	**—**	**1.40**
03-02-05	—	—	1.40	23-02-05	—	1	3.40
**06-03-05**	**—**	**—**	**10.00**	**20-03-05**	**1**	**1**	**0.91**
06-04-05	—	—	2.60	20-04-05	1	—	1.30
**08-05-05**	**1**	**—**	**1.30**	**22-05-05**	**1**	**—**	**4.00**

CuT + B/Total CuT	4/40	3/40	2.10 ± 1.40		3/40	3/40	1.40 ± 0.62

Midstream station							
04-08-04	—	—	1.40	16-08-04	1	—	4.90
**05-09-04**	**3**	**3**	**3.90**	**19-09-04**	**—**	**1**	**5.10**
07-10-04	—	1	1.40	22-10-04	1	—	0.71
**07-11-04**	**—**	**—**	**3.10**	**28-11-04**	**—**	**—**	**3.40**
16-12-04	—	—	1.20	22-12-04	—	1	1.80
**09-01-05**	**1**	**1**	**11.00**	**23-01-05**	**1**	**1**	**6.40**
03-02-05	—	—	8.80	23-02-05	—	—	4.90
**06-03-05**	**3**	**2**	**12.00**	**20-03-05**	**1**	**2**	**5.20**
06-04-05	—	—	3.30	20-04-05	3	1	1.40
**08-05-05**	**—**	**—**	**13.00**	**22-05-05**	**4**	**3**	**6.80**

CuT+ B/Total CuT	7/40	7/40	5.9 ± 2.3		11/40	9/40	4.0 ± 1.0

Downstream station							
04-08-04	1	—	8.10	16-08-04	1	1	4.50
**05-09-04**	**4**	**4**	**24.00**	**19-09-04**	**3**	**2**	**4.70**
07-10-04	2	3	12.00	22-10-04	—	1	0.95
**07-11-04**	**2**	**3**	**20.00**	**28-11-04**	**3**	**2**	**7.80**
16-12-04	—	2	17.00	22-12-04	—	—	3.10
**09-01-05**	**4**	**4**	**73.00**	**23-01-05**	**1**	**—**	**11.00**
03-02-05	4	4	33.00	23-02-05	—	—	7.80
**06-03-05**	**4**	**4**	**63.00**	**20-03-05**	**2**	**1**	**8.70**
06-04-05	3	3	31.00	20-04-05	4	—	1.80
**08-05-05**	**4**	**4**	**53.00**	**22-05-05**	**3**	**3**	**16.00**

CuT + B/Total CuT	28/40	31/40	33.00 ± 11.00		17/40	10/40	6.60 ± 2.20

ΣCuT + B/ΣTotal CuT	39/120	41/120			31/120	22/120	
ΣMean ± ΣSE of FC			13.00 ± 4.90				4.00±1.30

No. of CuT containing *Blastocystis* and mean ± SE of faecal coliforms during week day							
	10	13	8.20 ± 5.40		11	5	2.50 ± 1.00

**No. of CuT containing *Blastocystis* and mean ± SE of faecal coliforms during holiday**							
	**29**	**28**	**19.00 ± 11.00**		**20**	**17**	**5.50 ± 2.10**

CuT = culture tube, CuT + B = culture tube with positive *Blastocystis*, FC = faecal coliform, measurement in CFU/0.1L×10^3^, CFU = coliform forming unit, L = litre, SR = side of the river, MR = middle of the river, — = Nil, Σ = total, SE = standard error, **bold** = holidays.

**Table 4:** Correlations between *Blastocystis* and faecal coliforms, physical parameters, and rainfall in the two rivers.

Parameter	Sungai Congkak	Sungai Batu
Faecal coliforms	*r* = 0.80** *P* < .01, S	*r* = 0.50** *P* < .01, S
Dissolved oxygen	*r* = −0.30 *P* > .05, NS	*r* = 0.37* *P* < .05, S
pH	*r* = −0.35 *P* > .05, NS	*r* = −0.17 *P* > .05, NS
Temperature	*r* = 0.76** *P* < .01, S	*r* = 0.02 *P* > .05, NS
Conductivity	*r* = 0.19 *P* > .05, NS	*r* = −0.17 *P* > .05, NS
Turbidity	*r* = 0.30 *P* > .05, NS	*r* = 0.40* *P* < .05, S
Rainfall	*r* = −0.15 *P* > .05, NS	*r* = 0.24 *P* > .05, NS

*r *= correlation coefficients, *P *= probability level

S = significant, NS = not significant

**Correlation is significant at the 0.01 level (2-tailed)

*Correlation is significant at the 0.05 level (2-tailed).
